# Why do women want to be beautiful? A qualitative study proposing a new “human beauty values” concept

**DOI:** 10.1371/journal.pone.0201347

**Published:** 2018-08-03

**Authors:** Sunwoo Kim, Yuri Lee

**Affiliations:** 1 Department of Textiles, Merchandising, and Fashion Design, Seoul National University, Seoul, South Korea; 2 Research Institute of Human Ecology at Seoul National University, Seoul National University, Seoul, South Korea; Kyoto University, JAPAN

## Abstract

This study investigated the underlying reasons women desire to be beautiful in South Korean, Chinese, and Japanese cultures by proposing a new concept called human beauty value (HBV). This exploratory qualitative study includes a literature review in related disciplines and the results from ten focus group interviews. Based on the interviews, this study proposes four dimensions of HBV (i.e., superiority, self-development, individuality, and authenticity) and a hierarchical process among the antecedents (i.e., social comparison, social competition, and social norms), the pursuit of HBV, and the consequences (i.e., emotional, attitudinal, and behavioral aspects). Participants from each culture revealed a unique hierarchical process of HBV that reflects both cultural universality and specificity. The results of this study lead to new knowledge about East Asian women’s identities and perceptions of beauty. In addition, the proposed concept, HBV, can broaden the academic lens for beauty-related disciplines.

## Introduction

This study proposes a new concept, human beauty value (HBV), based on an exploratory qualitative study of women’s pursuit of beauty in three East Asian cultures: South Korea, China, and Japan. This study identifies the fundamental reasons women want to be beautiful by focusing on the socio-cultural phenomena that are relevant to women’s perceptions of beauty.

Beauty is only skin-deep, but the perceived absence of beauty may lead to damaging social bias. Compared to men, women may suffer more from social anxiety, prejudice, and inequality based on their appearance [1,2]. To comprehend beauty-related socio-cultural phenomena, many studies have examined the pursuit of beauty related to body image, body perception, a body-related self-concept, and body satisfaction [3–5]. These studies have clarified factors that impact and result in the pursuit of beauty. However, although numerous previous studies on these aspects of beauty have been conducted, there is still a considerable controversy about why women, in particular, are focused on with a beautiful appearance.

Modern East Asian women’s perceptions of beauty have not drawn enough interest in academia. Some studies have attempted to examine the diverse issues related to East Asian women’s perceptions of beauty, but they have collected data from only one culture or have considered the three distinct Asian cultures as the same cultural group [6–10]. As a result, these studies have not sufficiently identified women’s perceptions of beauty considering the cultural consistency and diversity of these three prominent East Asian cultures. Furthermore, the perceptions of beauty in modern East Asia have dramatically changed in a short period of time from the mid 20^th^ century to early 21^st^ century due to the cultural convergence between the cultural inheritance of East Asia and the inflow of Western culture due to industrialization and democratization [11]. As such, exploring East Asian women’s perceptions of beauty from a cross-cultural perspective can have substantial academic significance.

One of the purposes of social science is to manifest the underlying reasons why specific social phenomena occur by developing a theory explaining the relationship among socio-cultural antecedents, phenomena, and consequences, and ultimately predicting future conditions using the theory [12]. Based on this purpose of social science and the lack of previous effective research on East Asian women’s perceptions of beauty, the following objectives were developed:

1. Identify the fundamental reasons for women’s perceptions of beauty among three East Asian cultures: South Korea, China, and Japan.
2. Understand the hierarchical process among the antecedents, phenomena, and consequences related to women’s desire for beauty in three East Asian cultures: South Korea, China, and Japan.

To achieve these objectives, this study proposes the new concept, HBV to identify the ultimate value East Asian women put on beauty. The study also infers why they want to be beautiful. Additionally, this study explores the antecedents and consequences of these values and systematically attempts to understand the hierarchical process among the antecedents, the pursuit of HBV, and the consequences. To build a theoretical foundation of the concept, this study first defines HBV based on the limited available previous research. Then, to empirically demonstrate the concepts of HBV, we conducted an exploratory qualitative study.

### Conceptual definition and attributes of value

Since the definition of value is intuitive and ambiguous, it can be confused with other socio-cultural terms such as social norms, need, beliefs, or attitudes [13,14]. Due to this ambiguity, the meaning of value has often been modified depending on the purpose and discipline of the research. Consequently, diverse definitions exist [15]. Despite the various definitions, the definition by Rokeach [14] has been universally agreed upon [16]. According to Rokeach [14], “a value is an enduring belief that a specific mode of conduct or end-state of existence is personally or socially preferable to an opposite or converse mode of conduct or end-state of existence.” Different disciplines have adopted or adjusted this definition and developed their own research areas such as cultural value [17], lifestyle value [18], value in social adaptation and roles [19], consumer value [20,21], and social activity value [22]. Since different fields of research have developed customized definitions according to the purposes of the research area, many studies focusing on value have been conducted not only in specific research fields but also in diverse social science disciplines.

In numerous disciplines, the concept of value has taken on the roles of both independent and dependent variables in the study design [14,23]. Personal, social, and cultural factors have all received attention as antecedents of value, but the cultural factors have been regarded as the most crucial antecedent, especially in cross-cultural studies. [17]. Previous studies on the cultural antecedent of value have indicated that people in homogeneous cultures tend to pursue similar values [17,24]. Additionally, studies examining human values have argued that people in heterogeneous cultures share only a small number of values since there are only few core human values [25]. However, despite the similarity of essential values, each culture displays its own hierarchy of values depending on its unique cultural background [17]. The prevailing values in one culture are ones that are generally accepted among the members of the culture but may be distinctively different from values that are considered important in other cultures [26]. Thus, values affected by cultural characteristics contain both universal and special attributes, so using a cross-cultural approach would be an appropriate method to understand socio-cultural phenomena related to cultural values.

Another conceptual benefit of values is that we may infer cognition, emotions, and behaviors of individuals through the values they pursue. Since consequences of the perception of values has cognitive, emotional, and behavioral components [14], many previous studies on values have attempted to understand the psychological processes and socio-cultural phenomena of individuals. In the cognitive aspect, because values affect individuals’ decisions of what is desirable and the final end-state, they can generally evaluate people or events and eventually determine their attitudes based on their values [13]. These cognitive processes are accompanied by emotions toward pursuing the end-state and the related people and events, and these are the emotional consequences of values [14]. As for behavioral results, values affect the mode of conduct and set guidelines for behavior within a cultural context [27].

### Human beauty value

Based on the literature review above, this study proposes a definition of HBV and its attributes as follows. HBV is a value pursued through a focus on human beauty and a subjective belief related to a desirable or desired end-state of a beautiful body. It is also applied to modes of behaviors that control body conditions, which, in turn, guide the selection or evaluation of behaviors, people, or events involved in determining human beauty. HBV is a cultural product affected by socio-cultural contexts, and similar aspects of HBV could exist in various cultures. However, these values could be hierarchically and distinctly ordered depending on cultural characteristics. According to the aforementioned definition and attributes, this research investigates the following research questions:

RQ1. Verify the empirical existence of HBV in three East Asian cultures: South Korea, China, and Japan.
RQ2. Identify socio-cultural drivers affecting the pursuit of HBV in three East Asian cultures: South Korea, China, and Japan.
RQ3. Explore the consequences of the pursuit of HBV in three East Asian cultures: South Korea, China, and Japan.
RQ4. Comprehend the hierarchical process of antecedents, the pursuit of HBV, and consequences in three East Asian cultures: South Korea, China, and Japan

First, we propose HBV as a new concept through an empirical verification of HBV in three East Asian cultures. Second, using a cross-cultural approach, this study identifies the socio-cultural factors that motivate individuals to pursue HBV. We especially focus on the cultural universality among these three cultures and specificity the individual socio-cultural factors in each culture to understand the core factors of cultural distinctiveness in HBV. Third, this study explores the consequences of HBV from three perspectives: emotional, attitudinal, and behavioral. These consequences are also examined through cross-cultural perspectives to illustrate the cultural diversity as a result of HBV. Lastly, this study investigates the hierarchical process among the antecedents, the pursuit of HBV, and the consequences in order to propose a structural framework of HBV. By meeting these goals, this study pinpoints what makes people, particularly women, focus on beauty. Additionally, this study seeks to understand East Asian women’s values related to beauty, which is analogous but idiosyncratic in East Asian cultural backgrounds.

## Methods

### Participants

The participants in this study included women between 20 and 33 years old from South Korea, China, and Japan. These cultures to share somewhat similar cultural backgrounds [28], but they have maintained independent political structures for more than two thousand years and have retained unique languages, history, and customs. According to the cultural values of Hofstede [17], these three cultures share cultural universality affected by their East Asian cultural history, but they also have cultural specificity influenced by the uniqueness of each culture. For this reason, these three cultures were selected to examine the cultural influences of HBV and East Asian women’s perceptions and values related to beauty. Additionally, the women’s perceptions of beauty sharply contrasts with that of men, and women’s social stress and anxiety about beauty is more severe than men [1,2]. Hence, conducting research on women’s HBV is more urgent than on men.

The participants were recruited through on-line posts and bulletin boards located at five colleges in Seoul, South Korea. All interviewees were selected through brief questionnaires about their majors and interest in appearance and beauty. This study selected participants with diverse majors to reduce bias, and recruited participants answering more than three points on a five-point Likert scale for their interest in appearance and beauty to ensure abundant data on women’s beauty perspectives. In addition, we checked for fluency in the Korean language of the Chinese and Japanese applicants for seamless communication because all interviews were conducted in Korean. Additionally, based on the study of Avner and Weihl [29], which presented criteria for recruiting participants for cross-cultural studies in a single culture, we prioritized recruitment of Chinese and Japanese applicants who had lived a short period of time in South Korea to reduce Korean culture acculturation influences. To summarize, all interviewees were university students who lived in South Korea, aged from 20 to 33 years old, had a variety of majors, and had at least a moderate interest in appearance and beauty. The average length of stay in South Korea was 32.92 months for Chinese participants and 21.71 months for Japanese participants. All Chinese and Japanese participants communicated fluently in Korean. Detailed information about the participants is presented in Table 1.

**Table 1:** Demographic information on participants.

South Korea					China						Japan					
No	FGI Group	Age	Marital Status	Major	No	FGI Group	Age	Marital Status	Major	Length of stay in S Korea (Month)	No	FGI Group	Age	Marital Status	Major	Length of stay in S Korea (Month)
1	1	23	Single	Nutrition	1	4	23	Single	Linguistics	7	1	8	20	Single	Korean Language	2
2	1	24	Single	Nutrition	2	4	23	Single	Anthropology	7	2	8	22	Single	Korean Language	2
3	1	20	Single	Clothing & Textiles	3	4	25	Single	Clothing and Textiles	60	3	8	22	Single	Korean Language	13
4	1	20	Single	Education	4	4	23	Single	Clothing and Textiles	7	4	8	26	Single	Asian History	17
5	1	20	Single	Philosophy	5	4	24	Single	Clothing and Textiles	15	5	8	27	Single	Korean Language	11
6	1	23	Single	Economics	6	5	24	Single	Korean Literature	20	6	8	25	Single	Korean Language	12
7	1	21	Single	Philosophy	7	5	23	Single	Koreanology	4	7	9	26	Single	Translation Studies	36
8	1	24	Single	Nutrition	8	5	27	Single	Business Administration	36	8	9	20	Single	Humanities	14
9	2	27	Single	English Literature	9	5	24	Single	International Management	20	9	9	25	Single	Korean Language	8
10	2	22	Single	Pharmacy	10	5	25	Single	Korean Literature	48	10	9	21	Single	Business Administration	9
11	2	21	Single	Chemistry	11	5	25	Single	International Management	39	11	9	25	Married	Art	10
12	2	21	Single	Pharmacy	12	5	21	Single	Economics	36	12	9	21	Single	Korean Language	3
13	2	22	Single	Clothing & Textiles	13	6	25	Single	Public Administration	45	13	9	27	Single	Nutrition	27
14	3	32	Married	Clothing & Textiles	14	6	25	Single	Biotechnology	16	14	10	29	Single	Korean Language	84
15	3	32	Single	Merchandising	15	6	26	Single	Clothing & Textiles	36	15	10	28	Single	Photography	60
16	3	33	Married	Merchandising	16	6	23	Single	Hospitality Management	24	16	10	28	Single	Hospitality Management	46
17	3	33	Married	Management	17	6	28	Single	Korean Literature	11	17	10	28	Single	Business Administration	15
18	3	33	Married	Management	18	7	30	Married	Japanese Literature	8						
					19	7	32	Married	Korean Literature	82						
					20	7	32	Married	Political Science	40						
					21	7	31	Single	Korean Literature	14						
					22	7	30	Single	Korean Literature	96						
					23	7	30	Single	Korean Literature	60						
					24	7	31	Single	Political Science	36						
					25	7	30	Single	Mechanical Engineering	56						

### Procedure

This study received approval of the Institutional Review Board (IRB) at Seoul National University to ensure adherence to ethical research standards. Both researchers and interviewees agreed to comply with research ethics through a signed informed consent form. The form included information regarding the research purposes and procedures, extent of use of the collected data, discard of data, participant compensation, protection of privacy, and rights of the participants including the right to withdraw from the research project and refuse to answer any interview questions.

We conducted a total of 10 focus group interviews (FGI) with 60 participants: three FGIs with 18 Korean interviewees, four FGIs with 25 Chinese interviewees, and three FGIs with 17 Japanese interviewees. FGIs can be more effective than in-depth, one-on-one interviews on research topics because more abundant data can be collected through group dynamics [30,31]. Group dynamics is accelerated when FGI participants regard the other participants as social beings who are able to co-construct meaning while in an FGI [32]. Many previous studies related to body issues have preferred to utilize in-depth, one-on-one interviews to protect participants’ privacy; however, we used FGIs since the advantages of group dynamics can be greater, especially for this culturally related topic. Since the participants of this study created strong social bonds through co-constructing the meaning of women’s ideal beauty and the pursuit of beauty in their own cultures, we were able to collect rich meaningful data.

All FGIs lasted about two hours and utilized a semi-structured interview format [33]. Table 2 lists the pre-established interview questions. All FGIs were recorded, with permission from the participants, and then transcribed for analyses. The field notes were also analyzed since they included non-verbal language cues that cannot be heard in the recordings [34].

**Table 2:** Interview questions.

Category	Questions
**The Standards and the Values of Women’s Perception of Beauty**	1. Why do you think the woman in this image (research stimuli) is beautiful? 2. Did you have your own standards when you selected “cultural beauty” and “personal beauty?” If you did, what were the standards? 3. (If cultural beauty and personal beauty are different) Were there any reasons to identify “cultural beauty” and “personal beauty” as different? 4. (If “cultural beauty” and “personal beauty” are the same) Were there any reasons to identify “cultural beauty” and “personal beauty” as the same? 5. How does beauty affect your life as a woman?
**Antecedents to Women’s Perception of Beauty**	6. (For “cultural beauty”) Why do people in your culture perceive the woman in this image as beautiful? 7. What social influences or special treatment do beautiful women have in your culture? 8. Did you have any personal experiences that affected your selection of “cultural and personal beauty?” 9. Do you have any social background, social events, or peers that affected your selection of “cultural and personal beauty?”
**Consequences of Women’s Perception of Beauty**	10. How do you usually think about your body? 11. Were there any special behaviors, thoughts, or emotions that affected your perception of your body? 12. When you look at someone who is beautiful like the selected images, what do you think or feel about them? 13. Have you ever felt special emotions toward your body? If you have, what kinds of emotions have you felt? 14. When you get together with someone who is beautiful among your peers, what do you think or feel about them? 15. Do you make any special effort or have personal secrets to make yourself beautiful?
**Closing**	16. Is there anything else you would like to share or comment on?

### Stimuli

Creating the group dynamics in the FGIs was a very crucial issue that would determine the success or failure of this study. To facilitate the group dynamics, we used the stimuli method [34–36]. In East Asian culture where many people are afraid of losing face during communication [37], it is not easy to elicit group dynamics only through verbal communication. Lee and Lee [38] found that using stimuli in FGIs with East Asian participants could enhance group dynamics, and improve both the quality and quantity of the qualitative data.

In this study, images of beauty (beautiful women) were selected by the interviewees themselves were employed as stimuli. The images were classified as “cultural beauty,” which included characteristics that the public commonly perceives as beautiful in the culture of the interviewee, and as “personal beauty,” which included characteristics that the interviewee may personally perceive as beautiful based only on her personal aesthetic preference. Through the “cultural beauty” stimuli, we could identify the perception of ideal women’s beauty in each East Asian culture, and identify the cultural stereotypes and norms of women’s beauty. In contrast, through “personal beauty” stimuli, we could explore the ideal women’s beauty in the young adult generation in each culture. We also traced the changes in the preferences for ideal women’s beauty between young adult and traditional older generations by comparing the traditional “cultural beauty” stimuli and “personal beauty” stimuli.

Interviewees sent us images of women they perceived as beautiful via e-mail three days prior to the FGIs. There were no restrictions in choosing the stimuli so celebrities, models, or even interviewees’ acquaintances could be included. However, due to the time limitation of each FGI, the number of stimuli utilized per interviewee was limited to four: two for “cultural beauty” and two for “personal beauty.” All stimuli that had been selected by the interviewees were shared in the FGI in which the interviewee was assigned. Having interviewees provide the stimuli had a significant advantage to encourage group dynamics by breaking the ice and co-constructing meaning. Furthermore, the stimuli became a metaphor to guide diverse responses. These advantages were entirely consistent with a previous study that identified the visual stimuli that could be metaphors to induce underlying responses [39].

Table 3 presents detailed information on the stimuli. A total of 236 stimuli images were collected for the 10 FGIs, 116 for “cultural beauty” and 120 for “personal beauty.” For “cultural beauty” stimuli collected by the Korean and Chinese participants, there was a tendency to converge on two or three celebrities. Of the stimuli in the Korean FGIs, 53.85% identified actresses Kim Tae-Hee and Song Hye-Kyo as representatives of “cultural beauty,” and 52.17% of the stimuli in the Chinese FGIs identified actresses Fan Bingbing, Zhang Ziyi, and Liu Yifei as representatives of “cultural beauty.” For the Japanese participants, “cultural beauty” stimuli concentrating on specific celebrities was not as strong as for South Korean and Chinese participants. S1 File is the original data of the stimuli images and the result of frequency analysis of this data.

**Table 3:** Information on the stimuli of cultural beauty and personal beauty.

	South Korea		China		Japan	
	Name^a^	frequency^b^ (%)	Name	frequency (%)	Name	frequency (%)
**Cultural** **Beauty** **Images**	Kim Tae-Hee	14 (35.90)	Fan Bingbing	13 (28.26)	Matsushima Nanako	5 (16.13)
	Song Hye-Kyo	7 (17.95)	Zhang Ziyi	6 (13.04)	Kitagawa Keiko	5 (16.13)
	Lee Young-Ae	3 (7.69)	Liu Yifei	5 (10.87)	Ayase Haruka	3 (9.68)
	Kim Hee-Sun	3 (7.69)	Betty Sun	4 (8.70)	Sasaki Nozomi	3 (9.68)
	Jeon Ji-Huyn	2 (5.13)	Brigitte Lin	3 (6.52)	Nakama Yukie	2 (6.45)
	Others^c^	10 Celebrities (25.64)	Gao Yuanyuan	2 (4.35)	Shibasaki Kou	2 (6.45)
			Joey Wong	2 (4.35)	Others	11 Celebrities (35.48)
			Maggie Cheung	2 (4.35)		
			Vicki Zhao	2 (4.35)		
			Others	7 Celebrities (15.22)		
**Total(116)**	n^d^ = 15	39 (100)	n = 16	46 (100)	n = 17	31 (100)
**Personal** **Beauty** **Images**	Acquaintance	14 (34.15)	Acquaintance	24 (51.06)	Acquaintance	15 (46.88)
	Song Hye-Kyo	4 (9.76)	Ruby Lin	3 (6.38)	Kim Tae-Hee	2 (6.25)
	Son Ye-Jin	2 (4.88)	Son Ye-Jin	2 (4.26)	Others	15 Celebrities (46.88)
	Tang Wei	2 (4.88)	Zhou Xun	2 (4.26)		
	Sin Min-Ah	2 (4.88)	Others	16 Celebrities (34.04)		
	Others	17 Celebrities (41.46)				
**Total (120)**	n = 35	41 (100)	n = 43	47 (100)	n = 32	32 (100)

^a^ The name of beautiful women in the stimuli images.

^b^ The frequency of referring a specific woman’s image.

^c^ A total of one time mentioned stimulus.

^d^ The number of people referred to as the stimuli.

### Data coding and analysis

This study applied grounded theory (GT) for data analysis. GT is a qualitative research methodology to propose a new theory through the process of observations and interpretations of what people recognize and how they react to a specific phenomenon arising in the field [40,41]. In this context, theory refers to a framework composed of systematically connecting multiple concepts extracted from data collected from research participants on the complexities of their lived experiences in a social context [42]. Using GT, this study confirmed that HBV is a clear and present concept that exists in the three East Asian cultures. In addition, it identified sub-categories of HBV for clarification. Because GT can be applied in a variety of ways [42], we only applied the first and second steps from among the three analysis steps (i.e., open coding, axial coding, and selective coding). For open coding, we extracted categories of HBV, socio-cultural antecedents of HBV, and the consequences of HBV and then categorized these categories as main constructs. Next, for axial coding, we developed a paradigm model to demonstrate the integrated structure of HBV reflecting the mutual relationships among HBV, socio-cultural antecedents of HBV, and consequences of HBV. The paradigm model presented by Strauss and Corbin is an advanced and elaborate phase of analysis involving conditions, context, and consequences [40,42]. Accordingly, this study could systematically comprehend how HBV is formed and influenced by the socio-cultural contexts and which social consequences are derived through the paradigm model.

To verify the HBV concept and identify the framework of HBV, two authors participated in the analysis of the qualitative data collected from the 10 FGIs. The authors extracted units of text that conveyed one main idea related to HBV from the transcribed interviews and then categorized these units considering similarities in their meanings through the process of continuous and sustained comparisons. These initial categories were grouped into higher order categories that embraced the relevant sub-meanings, and finally, this study developed a hierarchical structure of categories and constructs related to HBV. Additionally, to reflect the cultural distinctiveness and universality of the three cultures in the framework of HBV, this study performed content analysis of the constructs and the main categories. The tested constructs were the HBVs, the antecedents of HBVs, and the consequences of HBVs, which were the results of the qualitative analysis in this study. The statistical procedures used in this content analysis included frequency distributions, cross-tabulations, and chi-square tests. The two authors held regular research meetings throughout this study from the research design to the final report in order to maintain consistency of the research plan, data collection, data analysis, and result interpretations.

## Results and discussion

This study confirmed that seeking HBVs is a social phenomenon in the three East Asian cultures. In addition, we identified socio-cultural antecedents and consequences of the HBVs as the highest constructs explaining the social phenomena associated with the HBVs. Table 4 illustrates the main constructs and categories and presents the results of the content analysis including frequency distributions, cross-tabulations, and chi-square tests. A total of six chi-square tests were conducted and all test results supported statistically significant differences in the frequency of each construct among the three cultures. S2 File is the frequency coding data for each category, and S3 File is the data for the chi-square tests.

**Table 4:** Qualitative construct, category, and frequency.

Construct	Category		n^a^ (%)^b^			Frequency^c^ (Mean)^d^		
			S. Korea (N = 18)	China (N = 25)	Japan (N = 17)	S. Korea (N = 18)	China (N = 25)	Japan (N = 17)
**Human** **Beauty** **Values**	Superiority		14 (77.78)	1 (4.00)	2 (11.76)	37 (2.64)	1 (1.00)	2 (1.00)
	Self-Development		7 (38.89)	22 (88.00)	14 (82.35)	13 (1.86)	79 (3.59)	29 (2.07)
	Individuality		6 (33.33)	11 (44.00)	16 (94.12)	6 (1.00)	17 (1.55)	65 (4.06)
	Authenticity		8 (44.44)	9 (36.00)	10 (58.82)	9 (1.13)	12 (1.33)	14 (1.40)
*X*^2^			31.926 (df = 6, p < 0.001)			193.886 (df = 6, p < 0.001)		
**Antece-dents**	Upward Social Comparison		12 (66.67)	4 (16.00)	12 (70.59)	25 (2.08)	4 (1.00)	22 (1.83)
	Introjection Comparison		11 (61.11)	16 (64.00)	3 (17.65)	22 (2.00)	24 (1.50)	3 (1.00)
	Projection Comparison		4 (22.22)	1 (4.00)	15 (88.24)	4 (1.00)	1 (1.00)	42 (2.80)
	Social Competition		14 (77.78)	8 (32.00)	11 (64.71)	66 (4.71)	10 (1.25)	22 (2.00)
	Social Norms		14 (77.78)	23 (92.00)	15 (88.24)	44 (3.14)	95 (4.13)	26 (1.73)
*X*^2^			33.858 (df = 8, p < 0.001)			198.286 (df = 8, p < 0.001)		
**Conse-quences**		Emotion: Ambivalent Emotion_Others	12 (66.67)	3 (12.00)	7 (41.18)	28 (2.33)	3 (1.00)	12 (1.71)
		Emotion: Ambivalent Emotion_Self	8 (44.44)	0 (0.00)	6 (35.29)	13 (1.63)	0 (0.00)	6 (1.00)
		Attitude: Positive Self Concepts	8 (44.44)	15 (60.00)	5 (29.41)	10 (1.25)	30 (2.00)	6 (1.20)
		Attitude: Negative Self Concepts	12 (66.67)	0 (0.00)	8 (47.06)	28 (2.33)	0 (0.00)	12 (1.50)
		Behavior: Body Modification	12 (66.67)	6 (24.00)	3 (17.65)	30 (2.50)	11 (1.83)	3 (1.00)
		Behavior: Body Supplement	10 (55.56)	19 (76.00)	16 (94.12)	39 (3.90)	53 (2.79)	66 (4.13)
*X*^2^			35.264 (df = 10, p < 0.001)			106.874 (df = 10, p < 0.001)		

Frequently mentioned categories in the three cultures are marked as highlighted cells.

^a^ The number of interviewees who referred to a specific category.

^b^ The percentage of interviewees who referred to a specific category.

^c^ The total number of times the interviewees mentioned a specific category.

^d^ The average number of interviewees’ responses referring to a specific category.

### Human beauty values

This study identified four dimensions of HBV (superiority, self-development, individuality, and authenticity). Through these dimensions, we could understand the desirable or desired perception of East Asian women’s physical beauty. From the findings, the authors could infer the reasons the participants in this study wanted to be beautiful and why they were focused on physical beauty in the context of different cultures.

### Superiority HBV

Superiority HBV means the pursuit of relatively superior competitiveness to surpass others with appearance and physical beauty. According to the superiority HBV, beauty could be classified into hierarchical levels, and the social competitiveness of appearance could be achieved only through the appearance of the superior level. This value was frequently mentioned in the Korean FGIs. As shown in Table 4, 14 (77.78%) Korean participants referred to the superiority HBV, with a total of 37 statements. Korean Interviewee 11 said, “There are levels in beauty. She is pretty, but she isn’t at a superior level. We could allocate a beautiful woman from the most beautiful level to just good or nice levels.” Korean Interviewee 16 talked about the Korean actress Kim Tae-Hee: “She could be popular because of the halo effect of Seoul National University. She is beautiful and even smart. So, game over. No one can beat her.” Korean Interviewee 6 said, “Beauty is a faultless woman perfectly approaching the ideal beauty, and they impress me with their outstanding appearance.”

We inferred that superiority HBV is a prevailing perception in South Korea due to the hypercompetitive society. Since social competition for limited resources and the social power of appearance has intensified in the past decade, Korean women have gradually believed that they could achieve more with limited social resources through superior beauty [43]. In fact, an attractive appearance has a positive impact on the evaluation of ability or personality based on the “halo effect” and people’s overall impression of others [44,45]. Previous studies have found that a beautiful appearance creates a competitive advantage that helps the individual acquire limited social resources such as an easier job search, the selection of a spouse, and higher income levels [46–48]. The male-dominant society in East Asia especially amplifies this phenomenon [49]. In this study, Korean participants frequently mentioned the gender discrimination in Korean families and communities. In reality, gender discrimination in South Korea has led to the objectification of women’s bodies and the intention to maximize advantages in social competition, even by going through risky appearance management such as cosmetic surgery [49].

### Self-development HBV

The self-development HBV is defined as finding meaning in the process of improving beauty through constant effort and management. According to this value, women can improve their beauty through personal endeavors. Women’s beauty is also a barometer to estimate how much effort she exerts for self-development. Based on this perspective, it cannot be said that only young women who maintain their biological youth are beautiful, but older women can also be beautiful if they take care of their appearance. The self-development HBV was often mentioned in the Chinese FGIs. As shown in Table 4, 22 (88.00%) of the Chinese participants, made statements about the self-development HBV, with a total of 79 statements. Chinese Interviewee 9 stated that she discovered this value through self-management: “In China, elderly women are more respected when they manage their appearance and make an effort. If she takes good care of herself, we should respect her. Despite her old age, her beautiful image is a symbol of self-development that impresses us.” Chinese Interviewee 5 said, “Beauty is the result of effort. There isn’t anybody that is muscular at birth. (Pointing at a stimulus) She receives attention because she managed herself and made significant effort to be beautiful. I want to be a well-managed beauty like her.” Finally, Chinese Interviewee 13 mentioned, “Simply, it’s not a problem of being pretty or not being pretty. If I don’t take care of myself, I can’t do anything else.”

We speculated that the self-development HBV, the most crucial value in China, is related to the increased level of gender equality in Chinese society. In China, the level of gender equality has gradually improved more than in South Korea and Japan because of the one-child policy and the diffusion of intensified gender equality policies after the post-Mao era [50,51]. The Chinese government had implemented the one-child policy to prevent a population explosion. Since Chinese families could have only one child legally, they had changed their preference for boys and now girls are cherished as well. This generation of girls can receive an equal education level as boys, and thus gain opportunities to have decent jobs [50]. Additionally, the Cultural Revolution by Mao, which refined the role and status of women by providing fair social opportunities and promoting social activities without gender discrimination, gradually eliminated patriarchal traditions that were deeply rooted in China [51]. Patriarchy, which is one of the traditional cultural characteristics in East Asian Confucian cultures, is a family system that is usually avoided in modern society since women are generally recognized as empowered individuals [52,53]. Patriarchy especially leads to male-dominant societies that deprive women of their healthy body image and distorts social standards that influence women to modify their bodies [54]. Therefore, gender equality relieves much of the social pressure on women’s appearance and improves women’s identity to control their own bodies.

### Individuality HBV

The individuality HBV is the pursuit of women’s beauty through distinctive individuality. According to this value, since beauty could be displayed in diverse forms, the ability to discover the proper type of beauty considering one’s personal image and physical features is crucial for women’s self-perceptions of beauty. To achieve this value, both the attempt and the acceptance of diverse types of attractive images are necessary. The individuality HBV was frequently discussed in the Japanese FGIs. As shown in Table 4, 16 (94.12%) of the Japanese participants made statements reflecting the individuality HBV, with a total of 65 statements. In other words, this figure can be interpreted that 16 interviewees mentioned the Individuality HBV more than five times during the FGIs. Japanese Interviewee 4 mentioned a Japanese actress who had her own unique beauty: “Tanaka Miho looks good with short hair. Kimono is also the same. Because she has a great fashion sense, her image is pretty and mild. These images are very unusual, novel, and attractive.” Japanese Interviewee 15 said, “In Japan, there are many types of beauty, so, I can choose what I want. I think expressing one’s own unique individuality is beautiful.”

This study assumed that the prevalence of the individuality HBV in Japanese culture reflects characteristics of the post-modern society seeking diversity. Post-modernism, an ideological foundation that advocates anti-aesthetics, incomplete, uncertainty, pluralism, and deconstruction, has affected the aesthetic appraisal of Japanese modern society [55]. According to post-modernism, beauty is appraised not by unchangeable or objective criteria but by flexible or subjective criteria that fluctuate depending on the circumstances [56]. For example, the art world, influenced by post-modernism, has even embraced ugliness such as kitsch, avant-garde, and the grotesque as aesthetic objects [55]. In this study, diverse socio-cultural criteria for the evaluation of women’s beauty increased the desire for the individuality HBV because that value encourages individual uniqueness. We infer that Japanese culture, as the pioneer to accept the modernized culture through the Meiji Restoration period, may concentrate more on individual unique beauty and show typical characteristics of post-modern society than the two other Asian cultures [57].

### Authenticity HBV

The authenticity HBV stresses that natural beauty is not about being decorated or imitated artificially. Women in the three cultures stated that the authenticity HBV was not achieved through artificial methods like cosmetic surgery but authenticity is naturally accumulated through life experiences. The authenticity HBV was universally mentioned in all three culture FGIs. However, compared to the previous three values, its importance was less significant. Korean Interviewee 7 mentioned the authenticity HBV: “I think one’s own image or attractiveness is more important, and these things aren’t acquired through cosmetic surgery because this beauty comes from one’s own ways.” Chinese Interviewee 5 mentioned the authentic beauty of Chinese actress Tang Wei: “All actresses have a pure or innocent image. But, Tang Wei has a special aura to be the only one in the world. It isn’t a fake image but her original one.” Japanese Interviewee 2 also talked about authentic beauty: “Real beauty can’t be verbalized. There is something special. This beauty is felt through one’s own beauty.”

Authenticity is receiving renewed attention as a way to resolve social problems caused by a hypercompetitive society [58,59]. Although authenticity can be defined differently depending on the purpose of the study, all the definitions include being genuine, trustworthy, and real. Authentic beauty unifies the inner self and the outer self by establishing a coherent identity [58,60]. The reason the three cultures identified authenticity as a HBV is that they must cope with the physical and psychological stress produced from the anxiety of appearance and negative social effects of “lookism.” Therefore, the pursuit of the authenticity HBV to accept women’s authentic self is a phenomenon to seek healthy beauty in both women’s inner and outer selves.

### Antecedents to HBVs

This study identified antecedents that influence the pursuit of HBVs. These antecedents, which were social comparison, social competition, and social norms, varied according to the cultural contexts of the three cultures. The details are as follows.

### Social comparison

Festinger initially introduced the concept of social comparison [61]. Humans interact and compare themselves with members of society in order to evaluate their own abilities or opinions that generate social power and opportunities. Social comparison is intensified when the judgment criteria for an ability are subjective, the achievement of an ability is especially challenging, or an ability is essential for socially important positions [61–63]. In this study, since the evaluation criteria of women’s beauty were subjective and women’s beauty was regarded as a way to facilitate a competitive social edge, the tendency of social comparison in appearance was confirmed in the three cultures.

The pursuit of HBV varied according to the diverse phenomena of social comparison. In the three cultures, the reference points and the estimated objects of social comparison revealed different patterns. These differences included their own particular social comparison phenomena, and these heterogeneous phenomena led to different pursuits of HBVs based on the culture of the interviewees. Additionally, the frequency of upward social comparison was a main driver to influence which HBVs the participants perceived as being significantly prevalent in each culture.

In Japan, the reference point of social comparison in women’s beauty was unique compared to Korean and Chinese cultures. In the process of social comparison for estimating the degree of physical beauty, Japanese interviewees did not react to the social standards that the social public considered to be ideal beauty. Rather, they tended to use a projection comparison by establishing themselves as a reference point. In contrast, the Korean and Chinese interviewees usually used introjection comparison by adopting social standards as a reference point. The difference between projection and introjection comparisons depends on the reference point and estimated objects [64]. In an introjection comparison, the reference points are others, and the estimated object is oneself. In contrast, in projection comparison, the reference point is oneself, and the estimated objects are others [65–67]. In this study, only Japanese women frequently used projection comparison. As shown in Table 4, 15 (88.24%) Japanese interviewees mentioned projection comparison, whereas only one Chinese and four Korean interviewees used projection comparison in the FGIs. This result corresponded to the study by Irmak, Vallen and Sen [64] who argued that the tendency of projection comparison is a characteristic of people seeking uniqueness. Japanese Interviewee 3, who stressed unique beauty to make herself stand out, commented on social ideals as estimated objects in social comparison of physical beauty: “Above all, it seems important to find a style that suits me. So, I have tried various things. I’ve read magazines, worn a lot of clothes, and tried diverse makeup styles.”

As for considering others as a reference point, the mass media seemed to strongly influence the social comparison of appearance as the standard of ideal beauty in the three cultures. As shown in Table 3, even though we did not restrict who participants could choose as stimuli (e.g., acquaintances or celebrities), only 53 cases (14 Korean, 24 Chinese, and 15 Japanese) of the 236 stimuli (80 Korean, 93 Chinese, and 63 Japanese) chose acquaintances as representatives of ideal beauty. This implies that the mass media substantially affects the formation of women’s perceptions of ideal beauty, which is consistent with previous studies that have identified the mass media such as fashion magazines, commercials, TV shows, and celebrities as the reference points of social comparison in appearance [68–70]. For Korean and Chinese cultures, there seemed to be strict stereotypes of their reference points since the stimuli of “cultural beauty” were mostly restricted to a few celebrities. As shown in Table 3, more than 50% of the Korean stimuli chose two popular actresses in South Korea, Kim Tae-Hee and Song Hye-Kyo, as representing “cultural beauty.” For the Chinese participants, 41.30% of the stimuli chose actresses Fan Bingbing and Zhang Ziyi as the top two most frequently mentioned “cultural beauty” representatives. However, the Japanese culture participants seemed to embrace individual beauty since they accepted more diverse criteria in their representative stimuli of “cultural beauty.” As shown in Table 3, only 32.26% of the Japanese stimuli identified celebrities, Matsushima Nanako and Kitagawa Keiko, as representatives of “cultural beauty” which was the lowest percentage among the three cultures. Additionally, a consensus on specific stimuli in Japan was lower than that of the other two cultures. Korean Interviewee 15, a big fan of a cosmetic surgery reality TV show, said, “I have watched the show not to miss any episodes. It was a real makeover. They were perfectly changed. I could do cosmetic surgery if I could change like them and only pay around $30,000 or $40,000.” This comment implies that the mass media had a strong impact on her social comparison in appearance as the standard of ideal beauty in South Korea.

In the Korean and Japanese FGIs, upward social comparison representing superior beauty as a reference point was frequently observed. As shown in Table 4, 12 Korean interviewees (66.67%) and 12 Japanese interviewees (70.59%) talked about upward social comparison, whereas only four Chinese interviewees (16.00%) mentioned it. Due to negative emotions such as envy, deprivation, and anxiety, people usually discontinue upward social comparison to maintain their mental health [71,72]. However, to have advantages in social competition, upward social comparison could be continued because of the desire to acquire such social ability [61]. Korean Interviewee 10 mentioned the comparison with a more beautiful friend: “I felt like I became her shadow. I made her more brilliant. She beat me even though she was my friend.” She expressed her negative experience caused by upward social comparison in appearance.

### Social competition

Social competition for women’s beauty was an antecedent to the pursuit of HBV as well. Social competition means holding a dominant position through interactive behaviors with social competitors to acquire limited resources [73,74]. In evolutionary psychology, social competition of appearance strengthens women’s desires for ideal beauty [75,76]. According to “The Origin of Species” [77], humans have evolved to transfer genes to future generations through sexual selection that regards the body condition of ideal beauty as excellent fertility [75]. Additionally, since women’s beauty has recently been considered a competitive advantage to create social power, a body that meets the social standards of a culture could achieve limited social resources [46,47].

In this study, social competition of appearance was more intense in South Korea and Japan. As shown in Table 4, 14 (77.78%) Korean interviewees and 11 (64.71%) Japanese interviewees mentioned social competition of appearance during the FGIs, whereas only eight (32.00%) Chinese interviewees mentioned it. Specifically, the average number of referrals per interviewee stating social competition in appearance in Korean FGIs was 4.71, which was nearly twice as high as in the other two cultures. These figures could be interpreted that the social power of beauty is stronger in South Korean and Japanese cultures than Chinese culture since Chinese interviewees believed that women’s ability was more crucial for social success than appearance. In contrast, the phenomenon of “social empowerment of beauty” is more prominent in Korean and Japanese cultures, implying that beautiful women have more social opportunities than less beautiful women. Korean interviewee 13, a master’s student who graduated from a prestigious university and has seven years of working experience in the fashion industry, said, “Actually, it was the reason why I had cosmetic surgery. When I started working, there were only a few employees that had a university-level degree. There were so many employees from a vocational school, but they were very pretty. When I saw them, I regretted that I went to school for such a long time and paid expensive tuition. They worked like me and made similar money. Why did I go through the hard time to graduate from a university?” Japanese interviewee 6 also said, “She was my friend in high school and was very pretty. Because I was her friend, I was able to benefit from her beauty. I was able to make many friends, and even better, go to stores and restaurants with her. But, I had an inferiority complex because of her at that time during appearance-sensitive puberty.” However, Chinese interviewees thought that discrimination against women’s appearance for social activities was unusual. Chinese Interviewee 3 replied, “In China, women’s ability and personality are more important for social success. If they are pretty, of course, it is good! But, women’s appearance doesn’t affect their success too much.”

### Social norms

Social norms related to women’s beauty influenced the pursuit of HBV as well. Social norms are the regulation of how social members think, including thoughts, language, behaviors, and perspectives [78]. In modern society, particularly, social norms that are related to appearance have been expanded through the mass media [68–70]. We inferred that South Korean and Chinese cultures have strict and powerful social norms regarding appearance since the “cultural beauty” stimuli were concentrated on a few famous celebrities, as shown in Table 3. This implies that celebrities’ appearances have a socio-cultural impact to unify the standards of ideal beauty, and these standards have become the social norms of ideal beauty. Furthermore, as shown in Table 4, 23 Chinese interviewees (92.00%) mentioned that social norms influence the standards of ideal beauty, with an average of 4.13 statements. In Korean FGIs, although there were slightly fewer interviewees than Japanese FGIs, the average number of times that Koreans mentioned social norms was still 3.14, which was higher than 1.73 for the Japan interviewees.

In South Korea, Confucian culture has restricted women’s gender roles and decisively affected social norms for women’s beauty compared to China and Japan where gender equality has gradually improved. Japanese Interviewee 2 said, “Recently in Japan, the birthrate is too low, so women can easily ask for maternity leave and return back to work.” This reply implies that gender equality has improved in the Japanese workplace. Chinese Interviewee 2 also discussed gender equality in Chinese families: “In China, both a husband and a wife usually have a job after marriage, so they divide the housework and do it together. Nowadays, Chinese men cook very well.” In contrast, in South Korea, although it is slowly changing, patriarchy has restricted women’s social roles, causing a vicious cycle that restricts women’s roles to domestic duties. In addition, in Korea’s patriarchy society, men have tended to concentrate on women’s appearance rather than on their abilities or personalities when choosing a spouse. Korean Interviewee 5 said, “Because Korea has been influenced by the Confucian culture for a long time, we prefer obedient women. So, soft and feminine images are popular.” Korean interviewee 14 also discussed women’s restricted social roles: “Because women’s social class could frequently change after marriage, women can succeed in life only if she gets married to a wealthy or high social class man.” Korean Interviewee 18 discussed the significant effect of women’s appearance on men’s choice for a spouse: “Matchmaking companies judge women only through appearance. Appearance might be 70 percent of the criteria to evaluate a woman. If she is tall, slim, and beautiful, she could be on a prestigious level.”

These restricted standards for women’s beauty affects the perception and pursuit of HBV. Most Korean women in this study preferred similar fashion and makeup styles and even preferred similar outcomes of cosmetic surgery. Japanese Interviewee 5, who had experienced the cultural differences between Korea and Japan while studying in South Korea, commented, “Korean women manage their appearance in a similar fashion with similar makeup and fashion styles, and even cosmetic surgery. When I first came to Korea, I was really surprised because all Korean women looked the same.” In contrast, Japanese culture accepted women’s diverse beauty. Japanese Interviewee 2 said, “I like different styles that I don’t have.” Another participant, Japanese Interviewee 5 mentioned, “Koreans like to have friends that have similar tastes or fashion styles. But, in Japan, it’s more frequent to have friends that have totally different styles.”

### Consequences of HBV

#### Emotions induced by HBV

The pursuit of HBV induced emotions that can be classified into two dimensions: emotions toward others whose bodies meet the socially ideal beauty standard, and emotions toward oneself whose body fails to fulfill social standards. Whereas previous findings have mostly focused on negative emotions, the interviewees in this study frequently mentioned ambivalent emotions regarding women’s beauty. Ambivalent emotions are defined as emotions that someone feels are both positive and negative, or they have more than two emotions toward the identical object, immediately or over a short time period [79,80]. Since ambivalent emotions are aroused when a person is under severe stress or anxiety [81], these emotions toward women’s appearance and beauty imply that women suffer from the social pressure of appearance. Because beauty basically arouses positive emotions such as aesthetic pleasure [82], when negative emotions including social pressure, stress, inferiority, and having a complex are combined with positive emotions, ambivalent emotions are eventually triggered.

In this study, Korean women seeking superiority HBV presented ambivalent emotions more frequently than Chinese and Japanese women. As shown in Table 4, 12 (66.67%) Korean interviewees mentioned ambivalent emotions toward others who had a beautiful appearance, and eight (44.44%) Korean interviewees mentioned ambivalent emotions toward themselves because they believed they did not reach the socially required ideal beauty standards. Korean interviewee 10 revealed her ambivalent emotions toward a beautiful friend: “My mother always told me not to be with her because I seemed like more ugly when compared to her. I knew, of course, but I wanted to be a friend with her because her pretty appearance made me happy. But, at the same time, I also felt inferiority. For a long time, I was in a strange state of feeling happy and inferior at the same time.” This result implies the severe stress and pressure related to women’s beauty as a result of competition in appearance.

In this study, emotions toward beautiful others were categorized into celebrities and peers. Both positive emotions (e.g., aesthetic pleasure, awe, and admiration) and negative emotions (e.g., sympathy, pity, and compassion) were simultaneously mentioned regarding celebrities who were exposed through the mass media. Korean Interviewee 1 said that she felt positive emotions rather than negative emotions when she met a newscaster: “When I met the newscaster, Oh Jung Hyun, I felt many emotions but not jealousy. She was a real doll. Wow! I just admired her. That’s all!” Japanese Interviewee 2 mentioned the emotions toward celebrities who managed their appearance in risky ways: “I felt pity. Why did they have to do? I thought the risky appearance management is not good for their health.”

Regarding beautiful peers, however, negative emotions such as jealousy, envy, and inferiority were predominant compared to positive emotions. This study inferred that this phenomenon may occur because the comparison appearance with beautiful peers had a more direct and powerful impact on the formation of interviewees’ negative self-concepts. Chinese Interviewee 16 stated, “If I had an extremely beautiful friend and she was popular with men, I could dislike her even if she’s my friend.” Korean Interviewee 6 felt inferior and an attraction toward her friend stating: “I felt inferior to a beautiful friend. Beauty is power. Frankly, pretty girls can receive a lot of comments on even boring posts on Facebook. I also want to be friends with a pretty girl. But, I felt very sad to realize I feel both inferior and attraction to her.”

#### Body image and attitudes toward human beauty

The perception and pursuit of HBV has influenced the perception of body image and women’s self-concepts. Many studies related to the psychological aspect of women’s bodies have explored the concept of body image, and defined it as humans’ perceived mental picture of their own bodies [83]. Since one’s body involves a sense of self, body image affects the development of one’s self-concept (e.g., self-esteem, self-efficacy, self-confidence, and self-discrepancy) beyond the body [5,44,84,85]. In this study, the Korean women with more ambivalent emotions toward beauty affected by the superiority HBV made more negative comments about body image and self-concept than Chinese women seeking the self-development HBV. As shown in Table 4, 12 (66.67%) Korean interviewees referred to their negative self-concept, whereas only non-Chinese interviewee referred to a negative self-concept. Additionally, compared to the Korean women, the Japanese women pursuing the individuality HBV claimed to have a healthier body image and self-concept due to the cultural acceptance of women’s diverse beauty characteristics, despite the high level of social competition in appearance. Eight (47.06%) Japanese interviewees talked about their negative self-concepts, and their average referral frequency was lower than that of the Korean participants (see Table 4).

Korean women who most frequently mentioned ambivalent emotions presented the most negative body image among the three cultures. Korean Interviewee 1 discussed her negative body image: “I really wanted to be like them. But I couldn’t. So, I blamed myself. If I were her, I wouldn’t need to lose weight every day. I could tie my hair with a ponytail, and I would be pretty when smiling. I really want to change my body.” However, they tried to maintain good mental health through the encouragement of a positive body image. Korean Interviewee 3 mentioned, “I like myself not because I am pretty but because I am who I am. Of course, I feel inferior and envy the beautiful people on TV.” To maintain a positive body image, an attempt to improve the shortcomings in appearance was also frequently discussed. Korean Interviewee 10 commented on maintaining a positive self-concept: “Because I knew I couldn’t be beautiful like her, I had to rather appeal to guys through my own attractiveness such as a good smile or my personality.”

Chinese interviewees who rarely expressed ambivalent emotions conveyed the most positive self-concept among the three cultures. As shown in Table 4, 15 (60.00%) Chinese interviewees discussed a positive self-concept, and their average referral was higher than the two other cultures. Chinese Interviewee 15 replied, “I am satisfied with what I have. So, I am glad and not greedy. I enjoy managing my appearance.” Chinese Interviewee 13 stated, “Beauty is just one of my advantages. Because I am a woman, I want to be beautiful through a clean and healthy appearance.” Japanese Interviewee 5 replied that she maintained a positive body image and self-concept by accepting differences between beauty and herself: “I also feel envious and inferior. But she and I are completely different, and I could have something that she doesn’t have. So, my effort to be beautiful is just enough for me.”

#### Behaviors to improve human beauty

The last consequence of seeking HBV observed in this study was behaviors related to managing appearance. In the three cultures, interviewees mentioned two approaches for their appearance management behaviors to improve their beauty: body supplements that are relatively safe methods such as fashion styling, makeup, skin care, and health care; and body modification that is risky such as cosmetic surgery and weight loss. This categorization is consistent with the study by Roach-Higgins and Eicher [86] classified appearance management behaviors according to the degree of persistence and risky effects. Body supplements are relatively less risky than body modification since these supplements are temporary decorations that display creativity, individuality, and aesthetic tastes through various applications [87,88]. In contrast, body modification could achieve ideal beauty through permanent or semi-permanent transformations in a short time [89]. However, body modification could result in side effects such as eating disorders (e.g., anorexia nervosa and bulimia nervosa), and psychopathological problems (e.g., body dysmorphia and cosmetic surgery addiction) [9,90,91].

In this study, Korean women seeking the superiority HBV preferred body modification to achieve radical results despite the potential risks because of hyper-competition of appearance. As shown in Table 4, 12 (66.67%) Korean interviewees mentioned positive consequences and their willingness to have body modification procedures even though it may put them at risk. Korean Interviewee 14 discussed the diffusion of cosmetic surgery: “It worried me that my sister wanted to get cosmetic surgery. She said young females think anyone who doesn’t get cosmetic surgery is an idiot.” Another Korean interviewee 10 talked about her experience of excessive weight loss: “I lost weight around 5 kilograms. At that time, I was really happy. For a while, I felt guilty when I ate until I was full and suffered from anemia and low blood pressure. After weight loss, I felt more self-confident, and I was able to wear whatever I wanted.”

Chinese women focusing on the self-development HBV mentioned health management, a topic Korean and Japanese women did not frequently discuss. Chinese participants believed that they could enhance their beauty by improving their mental and physical health. They regarded appearance management as only one of the ways to develop themselves and focused on self-oriented appearance management instead of social pressure. Chinese Interviewee 13 said that she could improve her appearance through having a healthy body: “I like exercising to manage my health. My major is just studying with a computer all day. Every early morning, I go to my lab and then come back to the dorm. So, I started to exercise at a gym last semester, and I also registered for a dance class this semester.”

Additionally, Japanese women pursuing the individuality HBV preferred body supplements to express their uniqueness. As shown in Table 4, 16 (94.12%) Japanese interviewees stated that body supplements were the way to make their appearance beautiful. They enjoyed appearance management to try diverse fashion styles as seen in this comment from Japanese Interviewee 15: “I’m enjoying it just as fun. I’m very happy to try diverse fashion styles.” Japanese Interviewee 1 sought to be unique through various fashion styles. She said, “I often read fashion magazines. When a style in a magazine is pretty, I follow that. When I read magazines, I can get more information of fashion.”

### The structure of HBV

#### Structural framework of HBV

The structural framework of HBV was identified by clarifying the inter-relations among the antecedents. The intensity of social competition related to the frequency of upward social comparison, and the strictness of social norms was strongly connected with the reference point of social comparison.

As a trigger for the pursuit of HBV, social comparison was relevant to social competition [92], and these two antecedents constituted the structural framework of HBV. As shown in Table 4, in South Korean and Japanese cultures, intense social competition and frequent upward social comparison of appearance simultaneously affected the HBV as the antecedents. Women in these cultures more frequently mentioned negative emotions toward beauty during upward comparison and were more competitive in working toward ideal beauty than Chinese women. These results are consistent with a previous relevant study by Gilbert, Price, and Allan who argued that humans would feel defensive emotions (i.e., envy, shame, defeat, depression, anxiety, and self-criticism) when they feel inferior during upward social comparison, and these emotions become a driver for increasing social competition to restore inferior self-concepts [93].

Another structural framework was the combination of the strictness of social norms and projection-introjection social comparison according to a reference point. As shown in Table 4, South Korean and Chinese interviewees were inclined towards introjection based on the strict social norms that were influenced by narrow social standards of ideal beauty, whereas Japanese interviewees were inclined towards projection that resulted from generous social norms and the acceptance of diverse beauty. These results correspond to findings of a previous study that social norms serve as reference points in social comparison [2]. When standards of reference points in social comparison are strict and narrow because of powerful social norms, the need for uniqueness decreases due to the desire to follow social stereotypes. For this reason, introjection whose reference point is not oneself but others is intensified, whereas the need for uniqueness is reinforced when social norms are not strict, so projection regarding oneself as a reference point is strengthened [64].

### The dimensions of HBV and the hierarchical process of HBV

**Fig 1:**
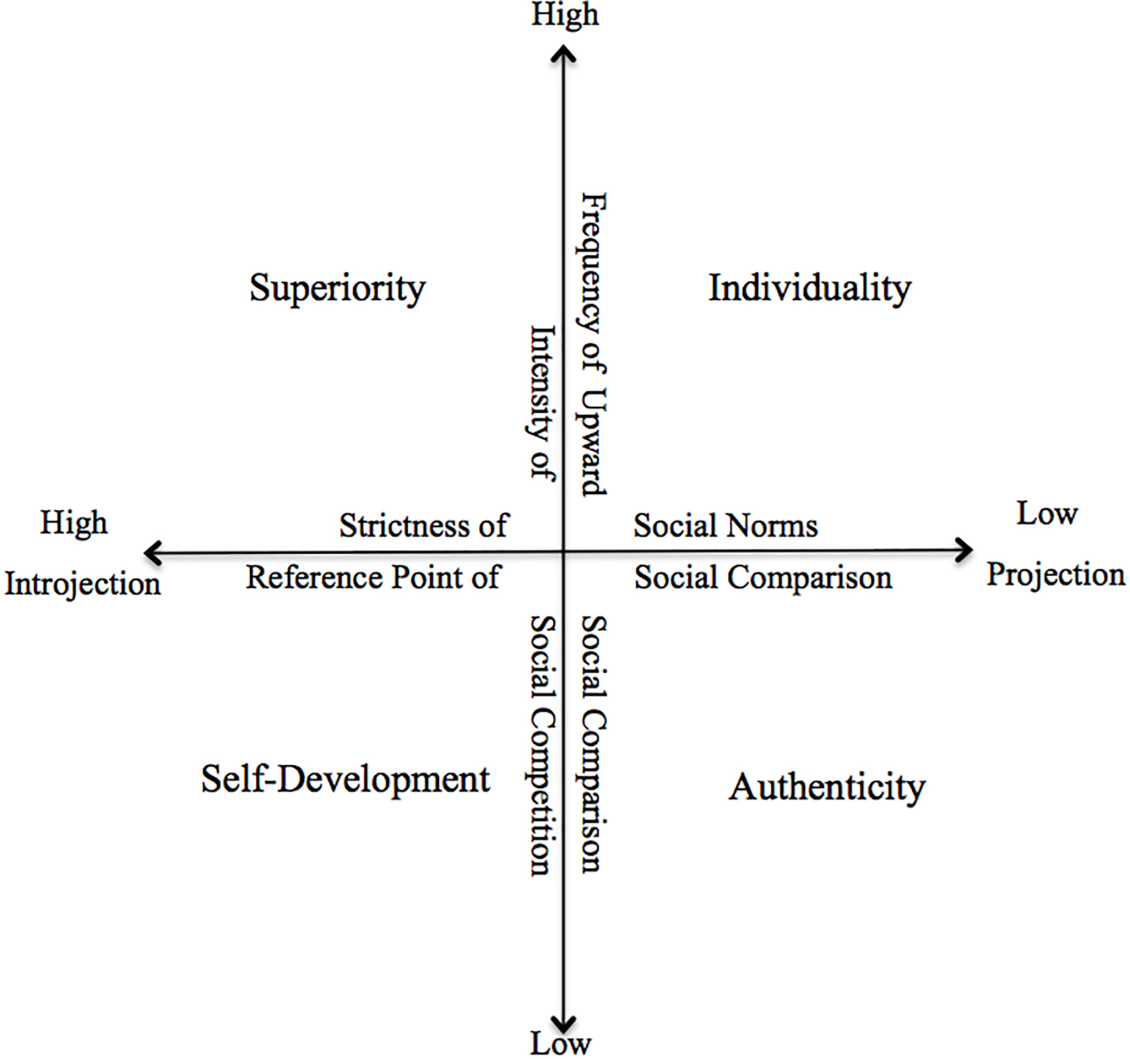
Structural framework and dimensions of HBV.

The structural framework of HBV revealed the dimensions of HBV, and this dimensional approach underpinned our understanding of the hierarchical process of antecedents, the pursuit of HBV, and consequences among South Korean, Chinese, and Japanese cultures. Fig 1 illustrates the structural framework and dimensions of HBV.

As shown in Fig 1, in South Korea, four socio-cultural antecedents affected the pursuit of HBV: intense social competition, frequent upward social comparison, strict and narrow social norms regarding women’s beauty, and introjection with ideal beauty as a reference point. According to these antecedents, the superiority HBV significantly prevailed in South Korea which reflects women’s desire to acquire social benefits through ideal beauty. In this regard, the consequences of the pursuit of HVB were the most negative and obsessive in South Korea among the three cultures. Ambivalent emotions were most frequently mentioned about both beautiful women having superior bodies and the self defeated in the battle of appearance competition. These ambivalent emotions led to a negative body image and self-concept. For appearance management behaviors, Korean women preferred risky methods that produced rapid and radical results such as cosmetic surgery or weight loss.

As shown to Fig 1, in China, the socio-cultural antecedents of introjection social comparison, strict social norms for ideal beauty, mild social competition in appearance, and infrequent upward social comparison influenced the pursuit of HBV. According to these antecedents, the self-development HBV was the most significant among the four HBVs in China. Although Chinese women wanted to follow social standards of appearance, they did not suffer from severe stress because of their appearance since Chinese culture accentuates not only women’s appearance but also women’s abilities. This phenomenon resulted in an emphasis on the self-development HBV through appearance management. As for emotional consequences, Chinese women usually felt positive emotions toward both themselves and beautiful women doing their best to manage their appearance, and they rarely felt ambivalent emotions. As for attitudinal consequences, their body image and self-concepts were more positive compared to South Korean and Japanese women. Finally, as for behavioral consequences, they were more self-motivated to engage in appearance management. Specifically, they improved their health to manage their appearance and regarded these behaviors as self-development.

In Japanese culture, the antecedents that affected the pursuit of HBV were diverse social norms in appearance, projection social comparison, intense social competition in appearance, and frequent upward social comparison. In line with these influences, Japanese women regarded the individuality HBV as the most crucial value. Despite intense upward social comparison and social competition in appearance, Japanese women attempted to demonstrate unique beauty since diverse types of beauty were socially adopted. Regarding the emotional consequences, the ambivalent emotions in Japanese culture appeared less than in South Korean culture but these emotions were more frequently mentioned compared to Chinese culture. As for attitudinal consequences, Japanese women’s perceptions of their body image and self-concepts were more positive than those of Korean women but were more negative than those of Chinese women. As for behavioral consequences, Japanese women preferred body supplement as a way to express their uniqueness, and they enjoyed these management behaviors with less anxiety.

Lastly, the authenticity HBV was observed in all three cultures. However, this value was not frequently mentioned compared to each culture’s prevailing value: superiority in Korea, self-development in China, and individuality in Japan. We expect that the authenticity HBV could be predominant in a culture where diverse standards of beauty are accepted and social competition in appearance is not significant. In other words, if a culture accepts each woman’s beauty and emphasizes women’s diverse characteristics such as her abilities and personality, women could develop their own authentic beauty without any social pressure or stress.

## Conclusion and future directions

This study proposed the concept of human beauty value (HBV) that could shed light on a more profound understanding of women’s perceptions of beauty in South Korean, Chinese, and Japanese cultures. To empirically clarify this concept, we conducted this exploratory qualitative research with East Asian women between 20 and 33 years old and suggested a structural framework, dimensions, and hierarchical process of HBV. The significant findings of this study are as follows.

First, this study systematically identified the homogeneity and heterogeneity in the perception of women’s beauty among three East Asian cultures. Whereas numerous preceding studies have considered these cultures as having similar cultural characteristics, this study verified that these cultures share cultural universality, but each culture shows cultural distinctiveness, particularly as it relates to women’s perception of beauty. In the hierarchical process among the antecedents, the pursuit of HBV, and consequences in the three cultures illustrated similar components, but each culture had its own dynamics. As a result, we have made one of the first attempts to understand East Asian women’s beauty in these diversified cultural contexts. This study could be extended to other cultural areas that are currently considered to have similar characteristics in the perception of human beauty.

Second, this study provides a starting point to predict diverse social phenomena related to women’s beauty by revealing the underlying reasons women pursue a beautiful appearance in each culture. We explored which types of beauty women idealized and why they wanted to be beautiful. We also investigated the pursuit process of HBV by investigating both socio-cultural antecedents and consequences of HBVs. Considering the purpose of social science to develop an academic theory to predict future directions, the verification of the pursuit process of HBVs can accelerate the prediction of social phenomena related to women’s beauty and appearance.

Third, the hierarchical process of HBV suggests a new paradigm toward studies related to women’s beauty from cross-cultural perspectives. We systematically investigated the effects of cultural universality and specificity. While previous studies examining cultural differences in women’s beauty have been fragmented focusing on only antecedents or consequences, the significance of this study lies in providing an integrated cross-cultural viewpoint in the perception of women’s beauty.

This study presents a theoretical framework for the concept of HBV and provides preliminary insights into the perception of beauty in three East Asian cultures: South Korea, China, and Japan. However, it is not without limitations. This study has representative problems of the sample as an exploratory qualitative approach and should be supplemented by future studies. The results of this study were drawn from a relatively small number of participants in focus group interviews. Only 60 women participated: 18 Korean, 25 Chinese, and 17 Japanese women. Therefore, it is difficult to generalize the results of this study because the collected data do not provide enough information to represent each culture’s general perceptions about women’s beauty. Thus, future studies should conduct quantitative analysis including parametric statistics with larger samples that could represent each culture.

Second, it is difficult to conclude that the Chinese and Japanese participants are perfectly representative of their home cultures due to potential acculturation effects influenced by Korean culture. All of the Chinese and Japanese participants were recruited from international students studying in South Korea. Considering this problem, applicants with the shortest length of stay in South Korea were selected first to reduce the acculturation effects [29]. However, we could not completely dismiss the acculturation effects of Korean culture. Indeed, many previous studies have found that the two cultural groups, residing only in their home cultures as well as experiencing other cultures, show cultural distinctiveness and universality at the same time [94,95]. Therefore, additional studies recruiting participants who reside in their home cultures should be conducted to alleviate this potential conflict.

It would also be beneficial to include male participants in future studies. Since men’s perceptions of beauty are different from those of women, the results of such a study may differ from what was found in this study. Through this suggested future research, we should identify the universality of the results in this study despite the gender differences. On the other hand, if different results are observed from male participants in future studies, it may have great implications that reveal the need for studies on men’s perceptions of beauty.

Lastly, confirmatory research supplementing this exploratory approach should be conducted. Initially, in order to establish the concept of HBV, a validated HBV scale should be developed. To develop such a scale, we should extract sub-categories in each dimension based on qualitative data and literature reviews of related studies and then implement statistical verification. An empirical investigation of the hierarchical process of HBV is also necessary. This study, which proposed a hierarchical process of HBV that reflected all phenomena regarding women’s beauty, explored the question, “Why do women want to be beautiful?” If future studies demonstrate these results using a quantitative approach, we can take a major step forward in developing new terminology.

## Supporting information

S1 File Stimuli images. (XLSX)

S2 File Data_Frequency analysis. (XLSX)

S3 File Data_Chi-square tests. (XLSX)
